# Virus-stimulated neutrophils in the tumor microenvironment enhance T cell-mediated anti-tumor immunity

**DOI:** 10.18632/oncotarget.9743

**Published:** 2016-05-31

**Authors:** Chin Yang Chang, Jiayu A. Tai, Sumin Li, Tomoyuki Nishikawa, Yasufumi Kaneda

**Affiliations:** ^1^ Division of Gene Therapy Science, Graduate School of Medicine, Osaka University, Suita, Osaka 565-0871, Japan

**Keywords:** HVJ-E, tumor associated neutrophils (TANs), anti-tumor immunity, tumor microenvironment (TME), cytotoxic T lymphocytes (CTLs)

## Abstract

The tumor microenvironment (TME) fosters tumors by attenuating anti-tumor immunity, reinforcing tumor cell survival and increasing angiogenesis. Among the constituents of the TME, here, we focused on tumor-associated neutrophils (TANs). First, we found that the combination of poly I:C and inactivated Sendai virus particles (hemagglutinating virus of Japan envelope; HVJ-E) synergistically suppressed tumor growth in the B16-F10 melanoma mouse model. In this model, poly I:C contributed to the recruitment of CD11b^+^Ly6G^+^ neutrophils to the TME, and co-injection of poly I:C and HVJ-E increased CD11b^+^Ly6G^+^FAS^+^ TAN in the TME. Depletion of neutrophils abolished the synergistic anti-tumor effect of HVJ-E and poly I:C in B16-F10 tumors. We revealed that C-X-C motif chemokine ligand 2 (CXCL2) is produced in the TME by poly I:C, but HVJ-E enhanced neutrophil infiltration of the TME does not occur. An anti-CXCL2 antibody inhibited the tumor suppression by HVJ-E+poly I:C. HVJ-E in combination with recombinant CXCL2 protein or CXCL2 pDNA suppressed mouse melanoma by increasing cytotoxic T lymphocyte activity against B16-F10 melanoma, which was abolished by an anti-Ly6G antibody. HVJ-E directly and indirectly increased FAS and ICAM-1 expression in cultured bone marrow-derived naïve neutrophils. Thus, HVJ-E activates anti-tumor immunity via anti-tumorigenic neutrophils in the TME. An HVJ-E vector containing the CXCL2 gene may be applicable as a novel cancer gene therapy strategy.

## INTRODUCTION

Current progress in cancer immunology elucidates the importance of immune checkpoint regulation [1]. Immuno-tolerance against cancers is achieved through the activation of immune checkpoint systems by cancer cells [2, 3]. A representative example is the expression of the PD-1 ligands, PD-L1 and L2, on the surface of cancer cells, which attenuates T cell function via PD-1 signal transduction to generate exhausted T cells [2, 3]. PD-1 ligands are expressed in both cancer cells and tumor-infiltrating immune cells, such as macrophages and dendritic cells [4, 5]. Moreover, cancers that express PD-L1 in immune cells are likely to be more aggressive than those exclusively expressing PD-L1 in cancer cells [5]. The immune cells are originally anti-tumorigenic, presenting tumor antigens and activating CD4^+^ and CD8^+^T cells [6, 7]. However, in the tumor microenvironment (TME), immune cells gradually change their properties from anti-tumorigenic to pro-tumorigenic [8, 9]. Under the hypoxic conditions of the TME, dendritic cells express PD-L1 [10]. Under aerobic conditions, lactate produced from cancer cells converts macrophages to pro-tumorigenic tumor-associated macrophages (TAMs) [11]. The TME also contains other cells, such as myeloid-derived suppressor cells (MDSCs), endothelial cells from tumor vessels and fibroblasts. MDSCs suppress the immune response against cancers through multiple pathways, such as the depletion of metabolites required for T cell function, production of reactive oxygen and nitrogen species to damage T cells, interference with T cell infiltration into the TME, the induction of regulatory T cells and promotion of M2 type TAMs [12]. TAM component cells produce indolamine 2, 3-dioxygenase (IDO) to deplete tryptophan, which results in T cell dysfunction [13]. Endothelial cells in the tumor vasculature express the FAS ligand, which kills the TME-infiltrating T cells that express FAS [14]. Cancer-associated fibroblasts (CAFs) produce CXCL12, which protects cancer cells against CD8^+^T cell attack [15]. Thus, the TME plays a variety of roles that support cancer cell survival and promote cancer progression by blocking anti-tumor immunity [16].

Recently, several studies have referred to the role of tumor-associated neutrophils (TANs) in stimulating tumor growth and suppressing anti-tumor immunity in the TME [17–23]. TAN supports tumor growth by secreting VEGF and MMP9, which promote angiogenesis [17]. Other pro-tumorigenic functions of TAN have been reported, such as tumor cell invasion via secretion of elastases, MMP8, MMP9 and cathepsin G [18] and tumor cell proliferation via COX-2-dependent prostaglandin E2 synthesis [19]. TANs, including many N2 type neutrophils, are immunosuppressive because tumor regression and CD8^+^ T cell activation are achieved by TGF-β blockade, which depletes N2 neutrophils [20]. Additionally, TAN secretes arginase-1, which dampens T cell function [21]. However, not all TANs are immunosuppressive. In the early stage of human lung cancer, TAN is not immunosuppressive; instead, it stimulates T cell responses [22]. Mishalian et al. observed that during mouse tumor progression, TANs gradually became pro-tumorigenic [23]. Therefore, TANs originally have the potential to suppress cancers in the TME [24].

Considering the contribution of the TME to the immune suppression anti-tumor response, successful immune therapy may be achieved by reconstructing the TME. We previously reported that the inactivated Sendai virus (hemagglutinating virus of Japan; HVJ) envelope (HVJ-E) exerts multiple anti-tumor activities. Direct intratumoral injection of HVJ-E activates anti-tumor immunity and induces direct cancer killing [25–27]. HVJ-E target cells in the TME include cancer cells, macrophages, dendritic cells, fibroblasts and endothelial cells [25–28]. As a result, HVJ-E can act as a modulator of the TME for cancer therapy. Although the anti-tumor activities of HVJ-E mainly depend on the retinoic acid inducible gene-I (RIG-I)/mitochondrial anti-viral signaling protein (MAVS) pathway, which recognizes viral RNA fragments in the cytoplasm, it has not yet been clearly demonstrated whether HVJ-E produces cytokines and chemokines independently of the TLR signaling pathway. Our hypothesis is that a more robust TME modulation may be created by combining a TLR agonist and HVJ-E as a novel cancer immunotherapy, especially if both materials synergistically enhance anti-tumor immunity by complementing each other.

## RESULTS

### Combination of HVJ-E and poly I:C synergistically enhances anti-tumor immunity in a mouse melanoma model

**Figure 1:**
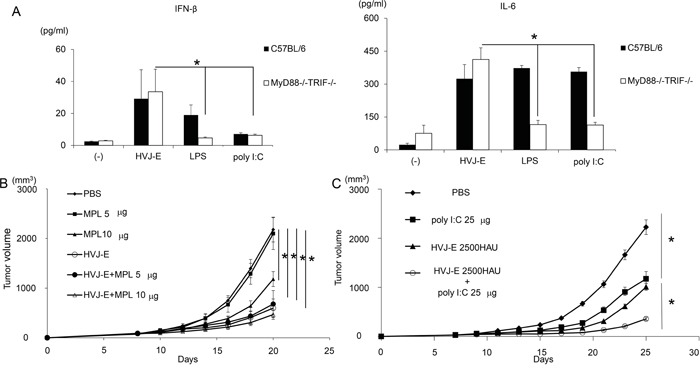
Anti-tumor effects of TLR agonists and HVJ-E **A.** IFN-β and IL-6 production from wild-type and Myd88−/−TRIF−/− mouse dendritic cells 24 hours after treatment with HVJ-E (1000 MOI), LPS (10 ng/ml) or poly I:C (50 μg/ml). Treatment of intradermal B16-F10 melanoma in mice with three injections of MPL (n=4). **B.** and poly I:C at 25 μg/mouse (n=4) **C.** with HVJ-E 2500 HAU. Significant suppression of tumor growth was achieved with the combination of HVJ-E and poly I:C (H+P), but not with HVJ-E and MPL, compared with single treatments. * Indicates p<0.05.

We chose two TLR agonists, lipopolysaccharide (LPS) and poly riboinosinic and poly cytidylic acid (poly I:C), which stimulate TLR4 and TLR3, respectively [29]. To examine whether HVJ-E works independently of TLRs, dendritic cells (DCs) from wild-type C57BL/6 mice and Myd88−/−TRIF−/− mice were treated with LPS, poly I:C and HVJ-E. As shown in Figure 1A, interferon (IFN)-β was produced by wild-type DCs in response to LPS treatment, but IFN-β was extensively suppressed in DCs derived from Myd88−/−TRIF−/− mice, which have no TLR downstream signaling. Poly I:C produced very low levels of IFN-β in wild-type and Myd88−/−TRIF−/− DCs. With either TLR agonist, IL-6 was produced by wild-type DCs, but it was not produced by Myd88−/−TRIF−/− DCs. However, HVJ-E treatment resulted in higher IL-6 and IFN-β levels produced by Myd88−/−TRIF−/− and wild-type DCs than the TLR agonists. We concluded that HVJ-E is independent of the TLR signaling pathway.

**Figure 2:**
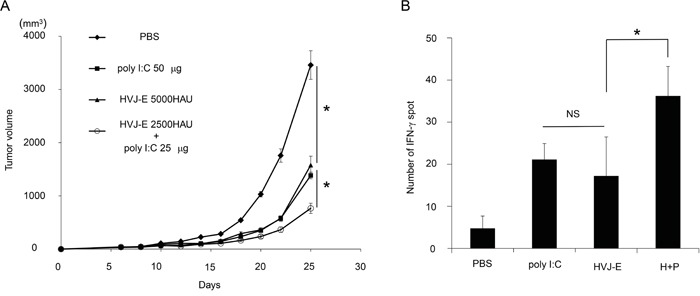
Synergistic anti-tumor effects of the combination of HVJ-E and poly I:C **A.** The combination of HVJ-E (2500 HAU) and poly I:C (25 μg) was more effective for tumor suppression than a double dose of HVJ-E (5000 HAU) or poly I:C (50 μg) (n=6). * Indicates p<0.05. **B.** Elispot assay for splenocytes. Splenocytes were isolated from tumor-bearing mice that were treated with 3 injections of PBS, poly I:C (25 μg), HVJ-E (2500 HAU) or HVJ-E+poly I:C (H+P) (25 μg+2500 HAU) 10 days after the last treatment (n=4). The numbers of IFN-γ-positive splenocytes stimulated with B16-F10 cells were counted. * Indicates p<0.05. NS indicates not significant.

For *in vivo* experiments, MPL, a modified LPS from S*almonella minnesota* [30], was used instead of LPS. We injected HVJ-E into mouse melanoma tissues with or without each of the TLR agonists, MPL or poly I:C. As shown in Figure 1B and 1C, HVJ-E, poly I:C and MPL all suppressed tumor growth, but the combination of HVJ-E with poly I:C, but not with MPL, demonstrated a greater reduction in melanoma growth compared with either HVJ-E or poly I:C alone. Then, the anti-tumor effects of the combination of HVJ-E and poly I:C were further analyzed. Based on the finding that the tumor suppression activity of poly I:C (25 μg) was comparable to HVJ-E (2500 HAU) (Figure 1C), the anti-tumor effects of the combination of HVJ-E (2500 HAU) and poly I:C (25 μg) were compared with those of HVJ-E (5000 HAU) and poly I:C (50 μg) (Figure 2A). The Elispot assay revealed that the number of B16-F10 melanoma cell-stimulated IFN-γ secreting splenocytes was significantly increased in mice that were treated with HVJ-E+poly I:C (36.2±7) compared with HVJ-E (17.2±9.2) and poly I:C (21.1±3.8) treatments (Figure 2B). These results suggest that HVJ-E and poly I:C may complement each other to enhance anti-tumor immunity.

### Neutrophil recruitment into the TME by CXCL2 contributes to tumor suppression by HVJ-E+poly I:C

**Figure 3:**
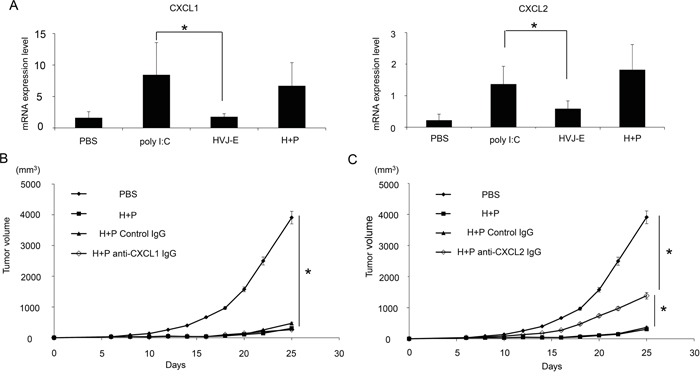
CXCL2 contributes to the synergistic anti-tumor effects of HVJ-E+poly I:C **A.** Expression of CXCL1 and 2 in the TME of B16-F10 melanoma in mice was assessed by qPCR (n=6). The effects of anti-CXCL1 (5 μg/mouse) **B.** or CXCL2 (50 μg/mouse) **C.** antibodies on tumor suppression with HVJ-E+poly I:C (H+P) (25 μg+2500 HAU) treatment were analyzed in melanoma-bearing mice (n=6). As a negative control, an isogenic antibody (Iso) was used. Antibodies were intratumorally administered three times 24 hours before HVJ-E+poly I:C injection and once 24 hours after the treatment (a total of four times). The tumor weight was compared between each treatment. * Indicates p<0.05.

Neither HVJ-E nor poly I:C alone suppressed the survival of B16-F10 melanoma cells *in vitro* (Supplementary Figure S1). To analyze the mechanism underlying this synergistic effect, we investigated cytokines and chemokines produced from melanoma tissues in mice injected with HVJ-E, poly I:C or a combination of HVJ-E and poly I:C (Supplementary Figure S2). We focused on the molecules that were detectable upon exposure to one reagent, either HVJ-E or poly I:C, compared with the negative control (PBS treatment). The results of the array were confirmed by qPCR. C-X-C motif chemokine ligand 1 and 2 (CXCL1 and 2) expression levels were significantly increased, compared with control levels, after poly I:C treatment, which was not the case after HVJ-E treatment (Figure 3A). In this array, no molecules were specifically enhanced by HVJ-E treatment. Next, to test whether either CXCL1 or CXCL2 was necessary for enhancing the anti-tumor effects of HVJ-E, an anti-CXCL1 or CXCL2 antibody was intratumorally injected into melanoma-bearing mice 24 hours before the injection of HVJ-E+poly I:C. The anti-CXCL2 antibody significantly abrogated the tumor suppression effects of HVJ-E+poly I:C, whereas the anti-CXCL1 antibody had no effect on the combination treatment (Figures 3B and 3C). Thus, we focused on CXCL2.

**Figure 4:**
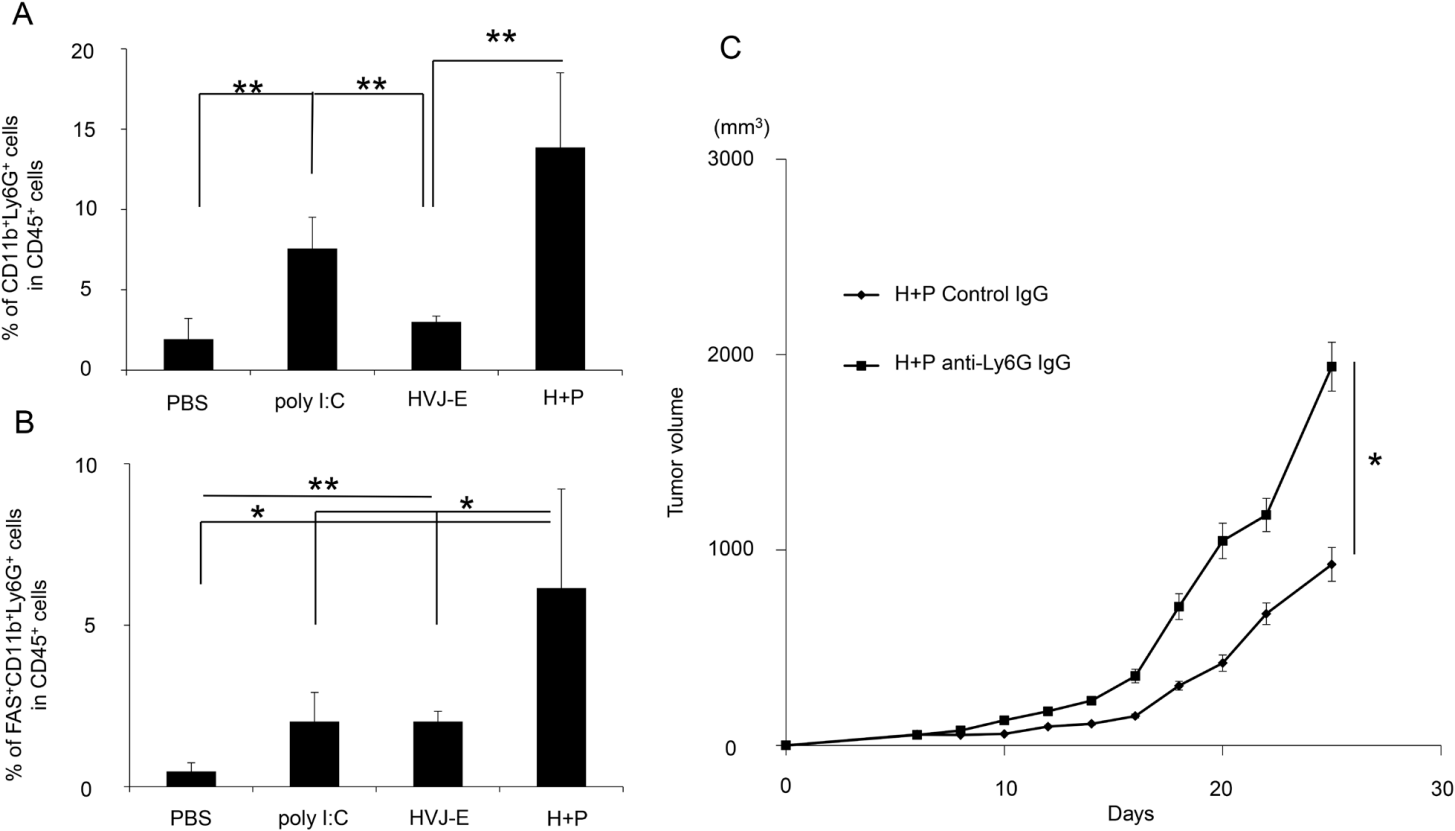
Accumulation and anti-tumor effects of neutrophils in tumor tissue **A.** To examine neutrophil accumulation in B16-F10 melanoma-bearing mice treated with PBS, poly I:C, HVJ-E or HVJ-E+poly I:C (H+P), the tumors were isolated after 3 injections with each reagent, and the ratio of CD11b^+^Ly6G^+^ cells to CD45^+^ cells in tumor tissues was analyzed using flow cytometry (n=4). **B.** To assess N1 neutrophil infiltration, the CD11b^+^Ly6G^+^FAS^+^ cell population was analyzed in tumor tissues (n=5). **C.** The effects of neutrophils on tumor suppression with HVJ-E+poly I:C were evaluated after the intratumoral administration of an anti-neutrophil antibody (50 μg/mouse) 24 hours before the combination treatment (n=6). ** Indicates p<0.01, and * indicates p<0.05.

CXCL2 is a chemoattractant for neutrophils [31]. Neutrophils that accumulate in tumor tissue are called TANs. Recent reports identified the following two types of TANs: anti-tumorigenic N1 and pro-tumorigenic N2 [20, 32, 33]. We analyzed the population of TANs (Figure 4A) and found that a CD11b^+^Ly6G^+^ population, which was identified as a TAN population [20], was more abundant in melanoma tissues that were treated with HVJ-E+poly I:C. Figure 4B shows that the CD11b^+^Ly6G^+^FAS^+^ population, which induces tumor-cell apoptosis, is an N1 neutrophil population [24] that was significantly increased in melanoma tissues treated with the combination therapy (6.1±3.1%) compared with HVJ-E (2±0.3%) or poly I:C (2±0.9%) treated therapies, whereas the expression levels of VEGF and MMP9, which are proangiogenic markers for N2 neutrophils [24], were decreased in CD11b^+^Ly6G^+^ neutrophils in response to the combination treatment compared with control (Supplementary Figure S3). The ratio of CD11b^+^Ly6G^+^FAS^+^ and CD11b^+^Ly6G^+^ICAM-1^+^ neutrophils per total neutrophil population was significantly increased by HVJ-E treatment compared with other treatments (Supplementary Figure S4). Then, the contribution of neutrophils to tumor suppression with HVJ-E+poly I:C was investigated. Both intratumoral (Figure 4C) and intraperitoneal (Supplementary Figure S5) administration of an anti-neutrophil antibody significantly inhibited the tumor suppression that resulted from the combination treatment. These results suggest that neutrophils recruited to the TME may be responsible for tumor suppression upon the HVJ-E and poly I:C combination treatment. We hypothesized that the combination of HVJ-E and CXCL2 might exert similar anti-tumor effects via anti-tumorigenic neutrophils in melanoma tissues. To test this possibility, we combined HVJ-E with CXCL2 recombinant protein instead of poly I:C.

### The combination of CXCL-2 and HVJ-E suppresses tumor growth by enhancing T cell-mediated anti-tumor immunity

**Figure 5:**
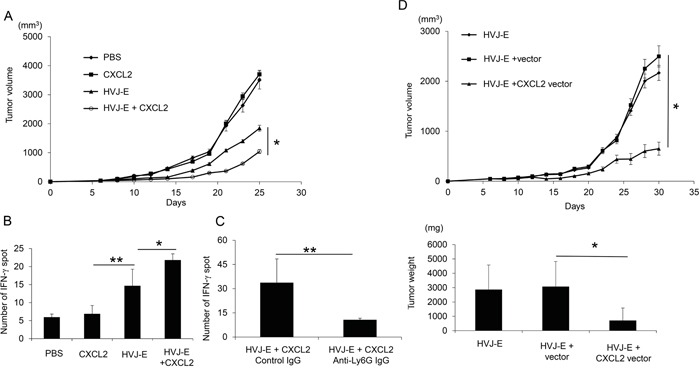
Enhancement of CTL activity against melanoma with the combination of CXCL2 and HVJ-E **A.** The anti-tumor effects of HVJ-E (2500HAU) combined with recombinant CXCL2 protein (0.3 ng/mouse) were evaluated in B16-F10 melanoma-bearing mice. The tumor volume on day 25 after tumor inoculation was compared for each treatment (n=6). * Indicates p<0.05. **B.** An Elispot assay with splenocytes against B16-F10 cells was performed (n=4). ** Indicates p<0.01, and * indicates p<0.05. **C.** The involvement of neutrophils in CTL activity against melanoma was examined. An anti-neutrophil antibody was intraperitoneally administered 24 hours before the intratumoral injection of HVJ-E+CXCL2 (2500HAU+0.3 ng/mouse) (n=4). Then, the Elispot assay with splenocytes against B16-F10 cells was performed. **D.** The effects of gene therapy using HVJ-E harboring a CXCL2 expression plasmid DNA (pCY4B-CXCL2) (50 μg/mouse) were examined in mouse melanoma. As a control, pCY4B without the CXCL2 cDNA (vector) was incorporated into HVJ-E. The tumor volume (upper graph) and isolated tumor weight (lower graph) on day 30 were measured (n=6). * Indicates p<0.05.

The novel combination of HVJ-E and recombinant CXCL2 protein resulted in increased tumor suppression compared with HVJ-E alone, whereas CXCL2 protein without HVJ-E did not suppress tumor growth (Figure 5A). Given that CXCL2 had no effect on the survival of B16-F10 melanoma cells *in vitro* (Supplementary Figure S6), the anti-tumor effects of the new combination treatment likely resulted from the enhancement of anti-tumor immunity. HVJ-E combined with CXCL2 resulted in increased IFN-γ expression in the Elispot assay, indicating an increase in cytotoxic T lymphocytes (CTLs) against B16-F10 melanoma cells (Figure 5B). However, the number of IFN-γ-positive spots in splenocytes in response to HVJ-E+CXCL2 (33.7±14.8) treatment was significantly suppressed with an anti-neutrophil antibody (10.7±1) (Figure 5C). These results support our hypothesis that anti-tumorigenic neutrophils are involved in the anti-tumor effects of the HVJ-E and CXCL2 combination therapy.

Because HVJ-E was originally developed as a gene delivery vector [33], we attempted to perform gene therapy against melanoma using an HVJ-E containing the CXCL2 gene. As shown in Figure 5D, CXCL2 gene therapy with the HVJ-E vector was more effective at suppressing melanoma in mice than HVJ-E alone or an HVJ-E-containing empty plasmid without CXCL2 cDNA. These results suggest that TANs recruited to the TME by CXCL2 may exert anti-tumor activity by polarizing the N1 type with HVJ-E.

### HVJ-E directly and indirectly stimulates the anti-tumorigenic properties of neutrophils

**Figure 6:**
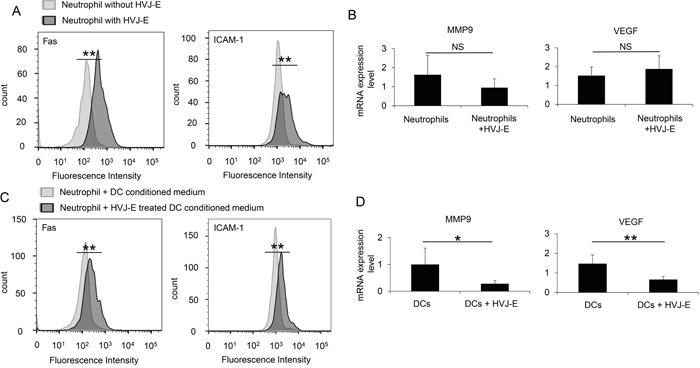
N1 polarization of HVJ-E-stimulated neutrophils **A.** FACS analysis of ICAM-1 and FAS expression in bone marrow-derived naïve neutrophils treated with or without HVJ-E (n=4). **B.** FACS analysis of ICAM-1 and FAS expression in bone marrow-derived naïve neutrophils that were cultured with conditioned medium from dendritic cells treated with or without HVJ-E (n=4). ** Indicates p<0.01. **C.** MMP9 and VEGF transcripts were assessed by qPCR in neutrophils (N) treated with or without HVJ-E (n=5). **D.** MMP9 and VEGF expression levels in neutrophils cultured with conditioned medium from dendritic cells (DC) treated with or without HVJ-E (n=5) were also determined. ** Indicates p<0.01, and * indicates p<0.05. NS indicates not significant.

Next, we analyzed the effects of HVJ-E on neutrophil properties. First, the direct interaction of HVJ-E with neutrophils was examined. As shown in Figure 6A, HVJ-E increased ICAM-1 (50.6±3.7%) and FAS (75.6±2.2%) expression in the neutrophil population, which are reported to be markers for anti-tumorigenic neutrophils [20, 23], compared with ICAM-1 (3.7±1.9%) and FAS (4.3±0.6%) expression in the control-treated neutrophil population. However, the expression levels of VEGF and MMP9, which are known pro-tumorigenic markers [24], were not altered (Figure 6B). Previous reports have revealed that DCs are HVJ-E target cells in the TME that are responsible for the HVJ-E-mediated activation of anti-tumor immunity [25, 26]. Based on these findings, neutrophils were incubated with a conditioned medium from DCs treated with or without HVJ-E. As noted in Figure 6C and 6D, ICAM-1 and FAS expression was significantly increased by the conditioned medium from HVJ-E-treated DCs, but VEGF and MMP9 expression levels were suppressed. The HVJ-E-treated DC conditioned medium significantly increased the ICAM-1 (54.2±0.6%) and FAS-positive (51.8±3.8%) neutrophil populations, whereas the untreated DC conditioned medium only elicited moderate ICAM-1 (10.6±3.8%) and FAS (10.4±1.9%) neutrophil populations. Thus, HVJ-E directly and indirectly enhances the N1 polarization of neutrophils.

### The combination of HVJ-E with poly I:C suppressed the growth of mammary carcinoma and colon adenocarcinoma in mice as well as mouse melanoma model

**Figure 7:**
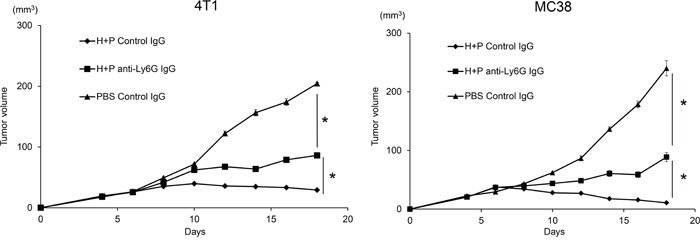
Combination of HVJ-E and poly I:C induced neutrophil-mediated anti-tumor effects in MC38 and 4T1 tumor models Mouse colon adenocarcinoma MC38 **A.** and mouse mammary carcinoma 4T1 **B.** cells were transplanted to C57BL/6 and Balb/c mice, respectively. On day 4 after tumor cell inoculation, tumors were treated with HVJ-E (2500 HAU) combined with poly I:C (25 μg) or PBS. For neutrophil depletion, the Ly6G (1A8) antibody or isotype control antibody was injected intraperitoneally (100 μg) three times 24 hours before HVJ-E+poly I:C treatment and once 24 hours after the last treatment (total of four times). * Indicates p<0.05.

To examine whether the combination of HVJ-E and poly I:C is effective for tumor suppression in various mouse tumor models other than mouse melanoma, we tested this combination treatment on 4T1 mammary carcinoma and MC38 colon adenocarcinoma tumor models. In Figure 7, combination HVJ-E and poly I:C treatment resulted in significant tumor suppression in both 4T1 and MC38 models, which was similar to that noted in the B16-F10 melanoma model. In these models, a neutrophil-neutralizing antibody attenuated the tumor suppression activity of the combination treatment. Therefore, the neutrophil-mediated antitumor activity of the HVJ-E and poly I:C combination can potentially be used in different tumor models, suggesting that neutrophils play an important role in antitumor immunity.

## DISCUSSION

Through cancer treatment using HVJ-E+poly I:C, we conclude that TANs are recruited by CXCL2 to exert anti-tumor activity in the TME in response to HVJ-E treatment.

It has been posited that TME reconstruction induced by altering the pro-tumorigenic phenotype of TANs and TAMs to the anti-tumorigenic phenotype may be more effective for enhancing anti-tumor immunity, as suggested by TGF-β blockade therapy [20]. Here, we demonstrated that HVJ-E altered the polarization of neutrophils to the anti-tumorigenic N1 type (Figure 6), which activated CTLs against melanoma (Figure 5). To the best of our knowledge, this is the first report to present evidence that neutrophil infiltration into the TME can enhance the adaptive immune response against cancer through exposure to HVJ-E. Our experiments indicate that neutrophils that are recruited to the TME by poly I:C or CXCL2 develop anti-tumorigenic properties in the presence of HVJ-E.

We demonstrated that HVJ-E directly and indirectly affected neutrophils via DCs. The indirect interaction was easily anticipated based on a previous report that HVJ-E-treated DCs activate NK cells via secreted IFN-β [26]. As shown in Figure 1A, IFN-β production in response to HVJ-E was considerably more effective than poly I:C. As the cytokine and chemokine array did not include type I IFN, we independently analyzed the production of IFN-β in the melanoma TME in the presence of HVJ-E, poly I:C or HVJ-E+poly I:C. IFN-β expression was increased in the TME treated with HVJ-E compared with poly I:C, but the difference was not significant *in vivo* (data not shown). This finding was presumably due to the instability of IFN-β in the TME. IFN-β contributes to the down-regulation of N2 markers [17]. The direct interaction of HVJ-E with neutrophils was unexpected because peripheral blood monocytes have no acidic ganglioside receptors for HVJ (data not shown), such as GD1a and sialylparagloboside, which are recognized by the HN protein of HVJ [34]. HVJ has two surface proteins, HN and F, and HN binds to acidic gangliosides. Then, F induces membrane fusion. However, we recently found that peripheral blood monocytes have no receptors for HN protein, but they fuse with HVJ-E, resulting in increased cytoplasmic calcium levels [35]. A similar signaling pathway may be activated in neutrophils through the interaction of the F protein with neutrophils. Given the importance of calcium signaling [36], F-protein-induced calcium signals may activate FAS and ICAM-1. Although the mechanism remains unknown, both direct and indirect interactions between HVJ-E and neutrophils may elicit N1 polarization of TANs in the TME. Thus, HVJ-E is likely a potent player in TME reconstruction because it can alter the pro-tumorigenic phenotype of TANs into anti-tumorigenic type cells.

Signaling pathways, including TLRs and RIG-I, have been extensively investigated, and similar transcription factors are activated by both pathways, such as IRF3, IRF7 and NF-κB [37, 38]. However, our results showed that the combination of HVJ-E with poly I:C, but not with MPL, synergistically enhanced anti-tumor immunity. Profiles of cytokines and chemokines activated by either HVJ-E or poly I:C did not completely overlap. It is hypothesized that the response to each agonist may differ in a variety of cells in the TME, including tumor cells, DCs, fibroblasts, endothelial cells, neutrophils and macrophages.

We identified CXCL2 as a chemoattractant to the TME for neutrophils [31] that was up-regulated by poly I:C but not by HVJ-E. Without HVJ-E, CXCL2 had no effect on tumor growth (Figure 5A). The combination of HVJ-E with recombinant CXCL2 protein or CXCL2-expressing plasmid DNA dramatically decreased melanoma growth via CTL activation against melanoma. However, the combination of HVJ-E+poly I:C appears to be slightly more effective for tumor regression compared with the combination of HVJ-E+CXCL2. Moreover, the anti-CXCL2 antibody could not completely abolish the tumor suppression achieved by HVJ-E+poly I:C treatment (Figure 3C). Thus, CXCL2 is not sufficient to achieve the anti-tumor activity observed with HVJ-E+poly I:C. These results suggest that poly I:C-induced factors in addition to CXCL2 may play a role in tumor suppression. Additionally, MPL could not induce CXCL2 expression and neutrophil infiltration to the tumor bed in a manner similar to poly I:C treatment (Supplementary Figure S7 and S8). This result showed that MPL combined with HVJ-E treatment could not induce synergistic anti-tumor effects via neutrophils that were similar to those noted for HVJ-E+poly I:C and further supports the fact that neutrophils play an important tumor suppression role in HVJ-E+poly I:C treatment.

It is surprising that inhibition of CXCL1, which is also a CXCR2 agonist similar to CXCL2 [31], exerted no inhibitory effects on the tumor suppression (Figure 3B) and neutrophil infiltration (Supplementary Figure S9) induced by HVJ-E+poly I:C treatment. Given that CXCL1 induces cancer growth and invasion [39, 40], the anti-tumor activities of HVJ-E+poly I:C may be cancelled by the pro-tumorigenic properties of CXCL1.

The FAS/FAS ligand pathway plays an important role in immune homeostasis by activation-induced cell death processes and the neutralization of host immune effects by tumor cells. FAS ligand expression on tumor cells could induce CD4 and CD8 T cell death [41] and neutrophil apoptosis [42] for immune escape. Fas is expressed by pro-inflammatory cells upon activation [41]. Therefore, high Fas expression on immune cells is not only a functional indicator for cellular apoptosis but also a marker for activated immune cells. This finding suggests that the activation of immune cells in the TME may not be sufficient and that the presence of a favorable environment in which these immune cells can survive is important for prolonging their effects on the tumor bed. From our results, poly I:C treatment increased ICAM-1 expression slightly more than PBS treatment, but not as high as the HVJ-E or HVJ-E+poly I:C treatments. Similarly, poly I:C also failed to increase Fas expression compared with HVJ-E or HVJ-E+poly I:C treatments. Although ICAM-1 is considered to be an N1 neutrophil [24], poly I:C treatment failed to induce neutrophil-mediated antitumor effect (Supplementary Figure S10). From this information, we hypothesize that although poly I:C treatment slightly increased ICAM-1 expression, this stimulation may be insufficient to provide an anti-tumor condition similar to the HVJ-E treatment. This finding provides strong evidence that HVJ-E is important to activate N1 polarization.

Furthermore, HVJ-E-stimulated IL-6 release may also play an important role in the neutrophil-mediated antitumor effects. A previous study demonstrated that IL-6 release induces a neutrophil-dependent anti-tumor response [43] and that IL-6 is important in helping neutrophils resist FAS pathway-induced apoptosis [44]. Previously, we demonstrated that HVJ-E induces DCs to release IL-6 [25] and exhibits other HVJ-E-induced antitumor effects. Taken together, HVJ-E stimulates neutrophil activation and may also exert a protective effect on activated neutrophils by increasing IL-6 levels in the tumor bed. Thus, neutrophils may induce more prolonged tumor suppression effects and increase T cell activation.

Through anti-cancer experiments, we propose that TME reconstruction is an effective cancer therapy approach, and the role of neutrophils and macrophages in the TME merits substantial attention. Our results demonstrate that HVJ-E combined with poly I:C or CXCL2 induced a neutrophil-mediated anti-tumor effect in various mouse tumor models, suggesting the potential wide spectrum of this anti-tumor mechanism. Because a clinical trial to treat melanoma patients using HVJ-E alone is ongoing in Japan, we are planning a novel gene therapy approach using the HVJ-E vector as the next step. CXCL2 may be a promising candidate for this purpose.

## MATERIALS AND METHODS

### Cell lines and mice

The B16-F10 melanoma cell line, 4T1 mammary carcinoma cell line and MC38 colon adenocarcinoma cell line were maintained in DMEM (Nacalai, Kyoto, Japan) with 10% FBS (BioWest, Nuaille, France) and 0.1 mg/ml penicillin-streptomycin mixed solution (Nacalai, Kyoto, Japan). Six-week-old, female C57BL/6N mice and BALB/c mice were purchased from Clea Japan. Myd88−/−TRIF−/− double knockout mice were constructed by mating Myd88−/− mice with TRIF−/− mice, which were kindly provided by Prof. Kiyoshi Takeda (Graduate School of Medicine, Osaka University, Japan). All mice were maintained in a temperature-controlled, pathogen-free room and were handled according to the approved protocols and guidelines of the Animal Committee of Osaka University (Suita, Japan).

### Virus

HVJ (VR-105 parainfluenza Sendai/52 Z strain) was purchased from the American Type Culture Collection (ATCC, Manassas, VA) and prepared as previously described [45]. Briefly, HVJ seed solution was injected into 10-day-old embryonated chicken eggs and incubated in a 37°C incubator for three days. After three days, allantoic fluid was harvested from eggs injected with HVJ. The recovered virus (live HVJ) was inactivated by UV irradiation (198 mJ/cm^2^) to form HVJ-E.

### MTS assay

Cell survival was detected using a CellTiter 96 Aqueous One Solution Cell Proliferation Assay kit (Promega, Fitchburg, USA). After dose-dependent treatment with poly I:C, HVJ-E or CXCL2, 100 μl of the CellTiter 96 Aqueous One Solution were added per 1 ml of medium. The absorbance was measured using a 96-well Mithras LB 940 Multimode Microplate Reader (Berthold Technologies GmbH & Co.KG, Bad Wildbad, Germany) at 490 nm.

### Tumor challenge treatment

Briefly, 10^6^ viable B16-F10 melanoma cells (in 50 μl of PBS) were intradermally injected into the backs of C57BL/6N mice. Six days later, when the tumor was 3 to 5 mm in diameter, the mice were intratumorally injected three times with HVJ-E (2.5 × 10^9^ or 5.0 × 10^9^ particles), poly I:C (25 μg or 50 μg) (Sigma, St. Louis, USA), or HVJ-E (2.5 × 10^9^ particles) combined with poly I:C (25 μg) or MPL (5 μg or 10 μg) in a total volume of 50 μl of PBS every 2 days. For MPL treatment, melanoma-bearing mice were treated with HVJ-E (1.0 × 10^9^ particles) and/or MPL (5 μg or 10 μg) (InvivoGen, San Diego, USA) as described above. The tumor volume was measured in a blinded manner using slide calipers and was calculated using the following formula: tumor volume (mm^3^) = length × (width)^2^/2.

### Tumor tissue cytokine and chemokine arrays and CXCL2 ELISA assay

Tumor tissues were collected from tumor-bearing mice 24 hours after the final treatment. The collected tissues were submerged in PBS and homogenized at 2500 rpm for 20 seconds using a Multi-Beads Shocker (Yasui Kikai Co. Osaka, Japan). After homogenization, Triton X-100 was added at a final concentration of 0.1%; protease inhibitors were also added. The samples were frozen at −80°C, thawed and centrifuged at 10,000 x g for 5 minutes to remove cell debris. Tissue lysates containing equal amounts of protein (400 μg) were subsequently used. A cytokine array was performed using cytokine array panel A (R&D Systems, Minneapolis, USA) according to the manufacturer's instructions. The results were analyzed using ImageQuant TL (GE Healthcare, Little Chalfont, UK).

For the CXCL2 ELISA assay, samples were prepared as described for the cytokine array. Mouse CXCL2 was assessed using an ELISA kit (DY452-05, R&D Systems, Minneapolis, USA) according to the manufacturer's instructions. The results were measured using a 96-well Mithras LB 940 Multimode Microplate Reader (Berthold Technologies GmbH & Co.KG, Bad Wildbad, Germany) at 540 nm.

### HVJ-E combined with recombinant CXCL2

PBS, HVJ-E and recombinant CXCL-2 (Biolegend, San Diego, USA) were injected three times (intratumorally) into B16-F10 melanoma-bearing mice every 2 days. After the final injection, the tumor size was monitored every 2 to 3 days. Tumors were collected and weighed on day 25.

### Plasmids and gene constructs

The murine CXCL2 gene was purchased from Sino Biological Inc. (North Wales, USA). The CXCL2 gene was amplified using iProof™ High-Fidelity DNA Polymerase (Bio-Rad) and the following primers:

Forward: 5′-AAGCTTGCCACCATGGCCCCTCCCACCT-3′

Reverse: 5′-CTCGAGTCAGTTAGCCTTGCCTTTG-3′.

The CXCL2 gene was cloned into the pCY4B vector for gene therapy experiments.

### *In vivo* gene therapy with the CXCL-2 expression vector carrying HVJ-E

In the *in vivo* gene therapy experiments, we used HVJ-E to transfer the CXCL2 expression plasmid (pCY4B-CXCL2) into B16-F10 tumors in C57BL6/N mice. Egg-derived HVJ-E was treated with a GenomeONE transfection kit buffer (GenomeONE; Ishihara-Sangyo Kaisha Ltd., Osaka, Japan). HVJ-E (2500 HAU) with pCY4B-CXCL2 (50 μg/50 μl per mouse) was injected three times into B16-F10 tumor-bearing mice (intratumorally) every 2 days. The tumor size was monitored until day 25.

### Preparation of splenocytes and dendritic cells

The spleens were harvested from C57BL/6N mice, and the cells derived from the spleens were filtered through a 40-μm mesh sieve and hemolyzed in hemolysis buffer (Immuno-Biological Laboratories Co., Ltd.). Mouse dendritic cells were isolated by flushing out the bone marrow of the tibia and femur with culture medium and then the samples were filtered through a 40-μm mesh sieve. After washing, the cells were cultured in a medium containing 10 ng/ml of recombinant mouse GM-CSF, as previously described [46]. Six days later, non-adherent and loosely adherent proliferating cells were identified as dendritic cells by evaluating CD11c expression using flow cytometry.

### Elispot assay

PBS, poly I:C and HVJ-E single and combined treatments were injected three times (intratumorally) into B16-F10 tumor-bearing mice every 2 days. The spleens were isolated from the mice 10 days after the last injection. Splenocytes were isolated from the spleens as described above. B16-F10 melanoma cells were treated with mitomycin C (15 μg/ml) for 45 minutes. The splenocytes and mitomycin C-treated B16-F10 melanoma cells were mixed at a ratio of 10:1. Forty-eight hours later, non-adherent splenocytes were collected, and an Elispot assay was performed using the Mouse IFN-gamma Development Module (R&D Systems, Minneapolis, USA) and the ELISpot Blue Color Module (R&D Systems, Minneapolis, USA). The numbers of IFN-gamma-secreting cells were subsequently counted.

### CXCL2 neutralization and neutrophil depletion

For the CXCL1/CXCL2 neutralization experiment, B16-F10 tumor-bearing C57BL6/N mice were pretreated with intratumoral injections of CXCL1 or CXCL2 neutralizing antibodies (R&D Systems) three times 24 hours before PBS, HVJ-E, poly I:C or HVJ-E+poly I:C treatment and one time 24 hours after the last treatment (total of four times). After the final injection, the tumor size was monitored every 2 to 3 days. Tumors were collected and weighed at the end of the experiments. For neutrophil depletion, the Ly6G (1A8) antibody was injected into the tumors (50 μg) or intraperitoneally (100 μg) a total of four times as described above. The tumors were collected and weighed at the end of the experiments.

### Quantitative real-time RT-PCR

Isogen (Wako, Osaka, Japan) was used to extract total RNA from tumors that were resected and washed in PBS. RNA was quantified, and 2 μg was used for reverse transcription into cDNA (Applied Biosystems). Quantitative PCR was performed using SYBR® qPCR Mix (Toyobo, Osaka, Japan) with primer sets for murine Ly6G, CXCL1, CXCL2, and β-actin (described below). The concentrations of the target genes were read using the CFX384 real-time system (Bio-Rad, CA, USA). All procedures were performed according to the manufacturer's instructions.

Ly6G

Forward: 5′-TGGACTCTCACAGAAGCAAAG-3′

Reverse: 5′-GCAGAGGTCTTCCTTCCAACA-3′

CXCL1

Forward: 5′-GACTCCAGCCACACTCCAAC-3′

Reverse: 5′-TGACAGCGCAGCTCATTG-3′

CXCL2

Forward: 5′-AAAATCATCCAAAAGATACTGAACAA-3′

Reverse: 5′-CTTTGGTTCTTCCGTTGAGG-3′

VEGF

Forward: 5′-GAGGATGTCCTCACTCGGATG-3′

Reverse: 5′-GTCGTGTTTCTGGAAGTGAGCAA-3′

MMP9

Forward: 5′-ACGACATAGACGGCATCCA-3′

Reverse: 5′-GCTGTGGTTCAGTTGTGGTG-3′

β-actin

Forward: 5′-GGAGGGGGTTGAGGTGTT-3′

Reverse: 5′-GTGTGCACTTTTATTGGTCTCAAG-3′

### Flow cytometry analysis of the tumors

Tumors were collected from mice and minced into fine pieces in a digestion buffer containing 2% FBS and 2.5 mg/ml collagenase A (Roche, Basel, Switzerland). Samples were incubated in the digestion buffer at 37°C for 1 hour with a shaker, filtered through a 70-μm filter, and washed twice with PBS. The collected cells were stained with the following fluorescent labeled antibodies: CD45 (30-F11), CD11b (M1/70), Ly6G (1A8), ICAM-1 (YN1/1.74) (BioLegend Inc. San Diego, USA) and FAS (eBioscience, San Diego, USA). All flow cytometry was performed on a BD FACSCanto™ II (Becton, Dickinson and Company, USA), and analysis was performed using FlowJo software (FlowJo LLC, Oregon, USA).

### Neutrophil isolation from tumor tissue and bone marrow

Tumor-associated neutrophils were isolated by magnetic bead separation according to the manufacturer's instructions (Miltenyi Biotec, Bergisch Gladbach, Germany). Tumors were collected from mice and minced into fine pieces in a digestion buffer containing 2% FBS and 2.5 mg/ml collagenase A (Roche, Basel, Switzerland). Samples were incubated in the digestion buffer at 37°C for 1 hour with a shaker, filtered through a 70-μm filter, and washed twice with PBS. Dissociated tumor cells were incubated with an anti-Ly6G biotin antibody (Miltenyi Biotec, Bergisch Gladbach, Germany) for 10 minutes on ice. After Ly6G antibody staining, cells were incubated with Anti-Biotin MicroBeads (Miltenyi Biotec, Bergisch Gladbach, Germany) for 15 minutes on ice. Cells were washed with PBS and sorted using an autoMACS separator (Miltenyi Biotec, Bergisch Gladbach, Germany). Mouse bone marrow neutrophils were isolated by flushing out bone marrow cells with a murine neutrophil isolation kit (Miltenyi Biotec, Bergisch Gladbach, Germany). Briefly, the flushed out bone marrow cells were incubated with the Neutrophil Biotin-Antibody Cocktail (Miltenyi Biotec, Bergisch Gladbach, Germany) for 10 minutes on ice. After incubation, the cells were washed with PBS and incubated with Anti-Biotin MicroBeads (Miltenyi Biotec, Bergisch Gladbach, Germany) for 15 minutes on ice. Cells were washed with PBS and sorted using an autoMACS separator (Miltenyi Biotec, Bergisch Gladbach, Germany). Sorted cells were analyzed with FACS, and the Ly6G-positive cell population was greater than 90%.

### Colon carcinoma and mammary carcinoma tumor challenge treatment

Briefly, 2 × 10^6^ viable MC38 colon adenocarcinoma cells and 4T1 mammary carcinoma cells (in 50 μl of PBS) were intradermally injected into the backs of C57BL/6N and BABL/c mice, respectively. Four days later, when the tumors were 3 to 5 mm in diameter, the mice were intratumorally injected three times with HVJ-E (2.5 × 10^9^ particles) combined with poly I:C (25 μg) or PBS. For neutrophil depletion, the Ly6G (1A8) antibody pretreatment was intraperitoneally injected (100 μg) three times 24 hours before HVJ-E+poly I:C treatment and one time 24 hours after the last treatment (total of four times). After the final injection, the tumor size was monitored every 2 to 3 days.

### Statistical analysis

Statistical analyses were conducted using Student's two-tailed unpaired t-test with GraphPad, and P-values less than 0.05 were considered statistically significant.

## SUPPLEMENTARY FIGURES


