# Supplementary material for: Virus-stimulated neutrophils in the tumor microenvironment enhance T cell-mediated anti-tumor immunity
Source: Oncotarget. 2016 May 31;7(27):42195–207. doi: 10.18632/oncotarget.9743 (PMC5173127; doi:10.18632/oncotarget.9743)
Supplement: Supplementary file 1 [file oncotarget-07-42195-s001.pdf]

Virus-stimulated neutrophils in the tumor microenvironment enhance T cell-mediated anti-tumor immunity

SUPPLEMENTARY FIGURES

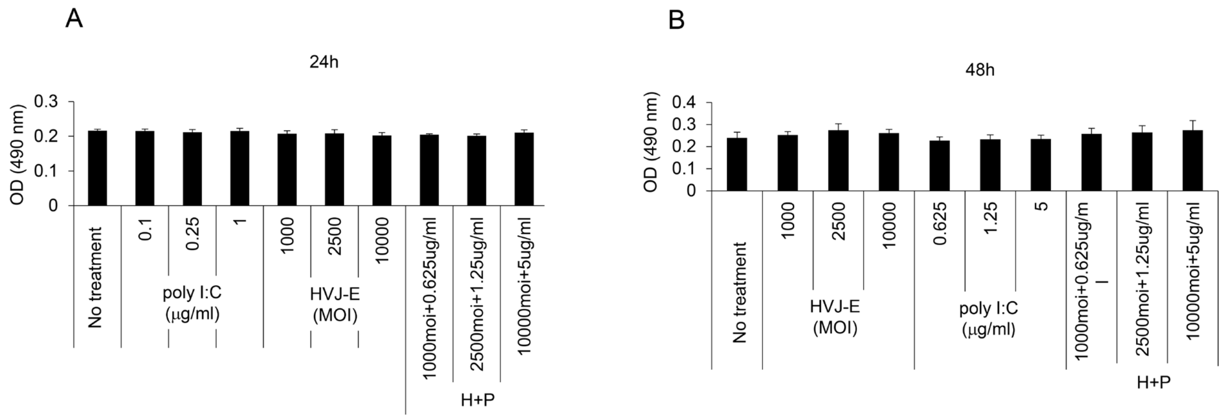

Supplementary Figure S1: B16-F10 cell survival was assessed with an MTS assay at 24 A. and 48 B. hours after treatment with various levels of HVJ-E, poly I:C or HVJ-E+poly I:C (H+P).

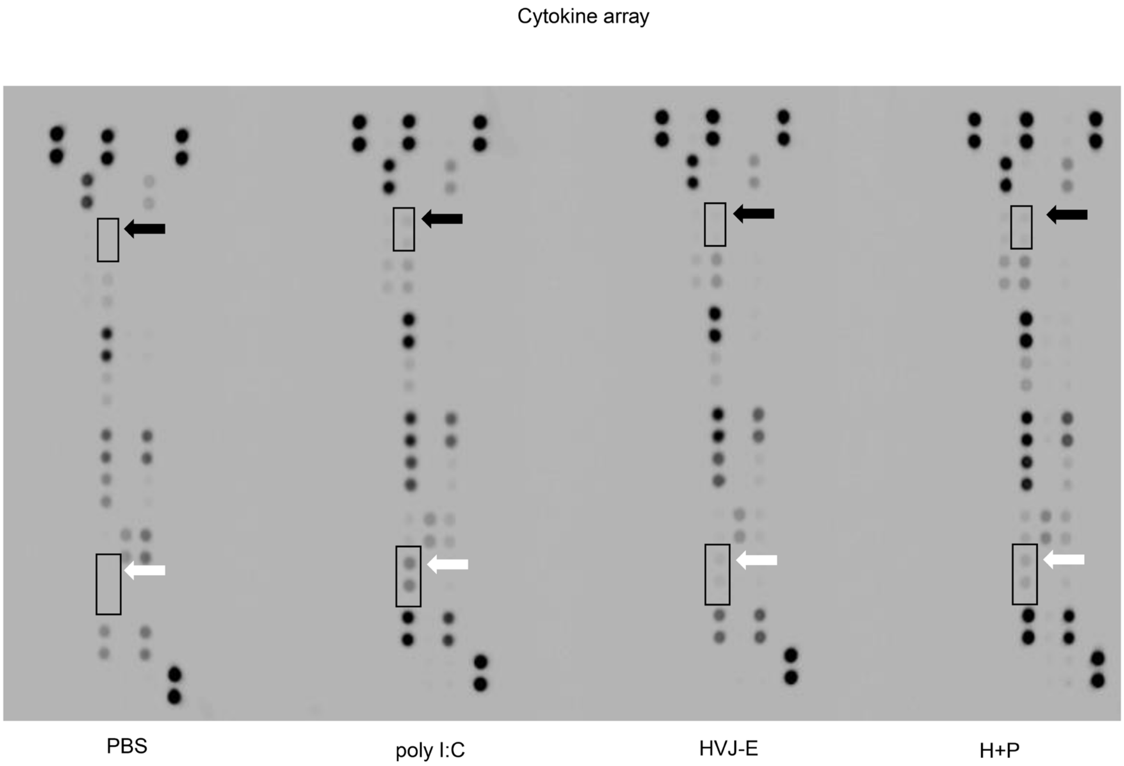

Supplementary Figure S2: Cytokine and chemokine array of B16-F10 melanoma tissues that were treated with PBS, poly I:C (25 µg), HVJ-E (2500 HAU) or HVJ-E+poly I:C (H+P) (25 µg+2500 HAU). Black and white arrows indicate CXCL1 and CXCL2, respectively.

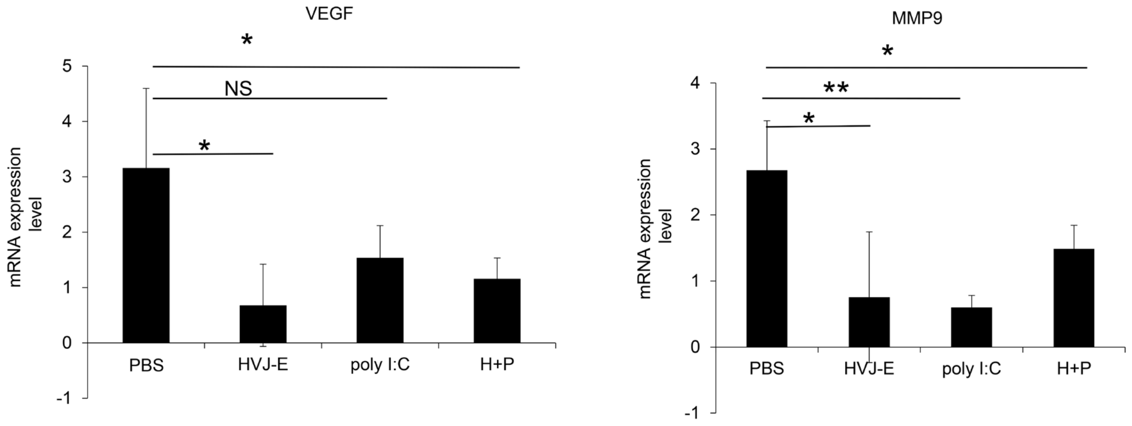

Supplementary Figure S3: VEGF and MMP9 expression levels were analyzed by qPCR in CD11b+Ly6G+ neutrophils that were isolated from tumor tissues treated with PBS, HVJ-E, poly I:C or HVJ-E+poly I:C. NS: not significant, *: $p<0.05$, **: $p<0.01$.

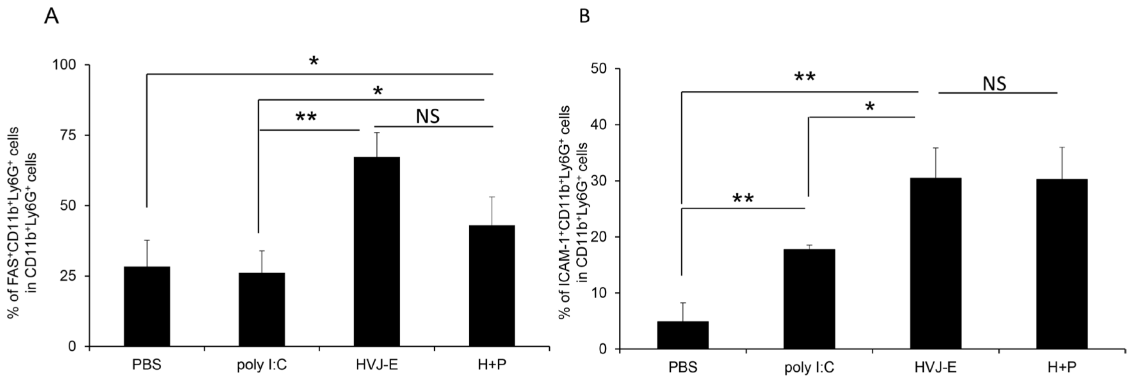

Supplementary Figure S4: The analysis of neutrophil character in B16-F10 melanoma treated with PBS, poly I:C (25 µg), HVJ-E (2500 HAU) or HVJ-E+poly I:C (H+P) (25 µg+2500 HAU). After three injections of each reagent, the ratio of $FAS^{+}CD11b^{+}Ly6G^{+}$ cells **A.** and ICAM-$1^{+}CD11b^{+}Ly6G^{+}$ cells **B.** to $CD11b^{+}Ly6G^{+}$ cells in tumor tissues was examined by flow cytometry (n=4). **; $p<0.01$.

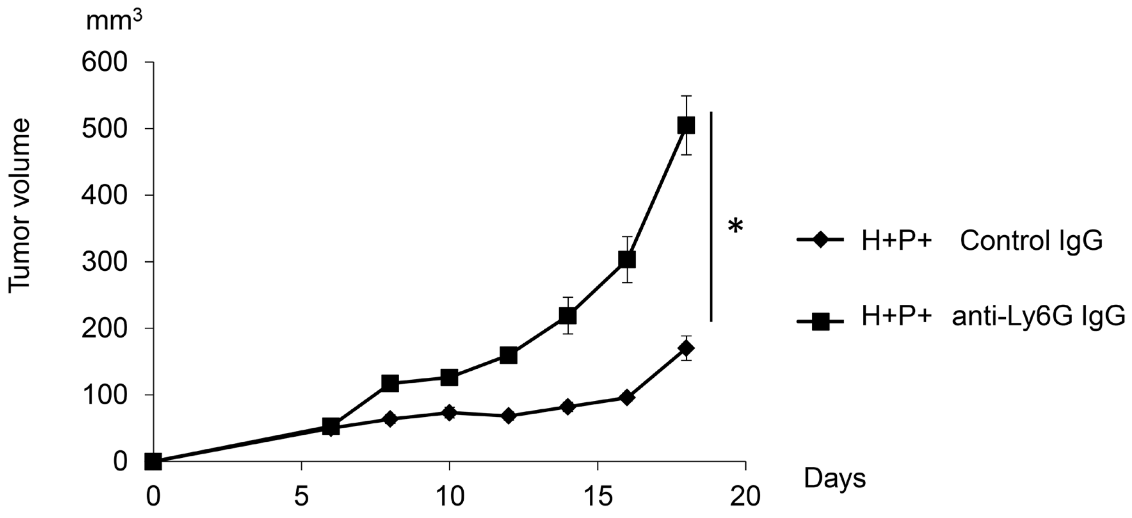

Supplementary Figure S5: The effects of neutrophils on tumor suppression with HVJ-E+poly I:C (H+P) (25 µg+2500 HAU) were evaluated by the intraperitoneal administration of an anti-neutrophil antibody (100 µg/mouse) 24 hours before the combination treatment.

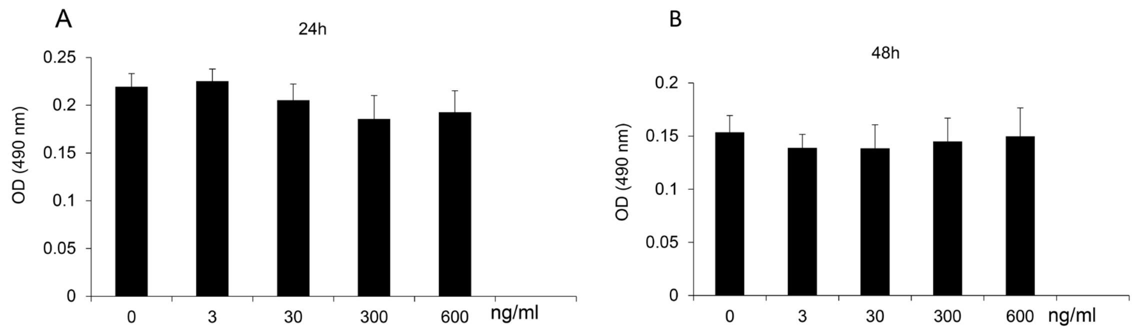

Supplementary Figure S6: B16-F10 cell survival was assessed using the MTS assay at 24 A. and 48 B. hours after treatment with various levels of recombinant mouse CXCL2 protein.

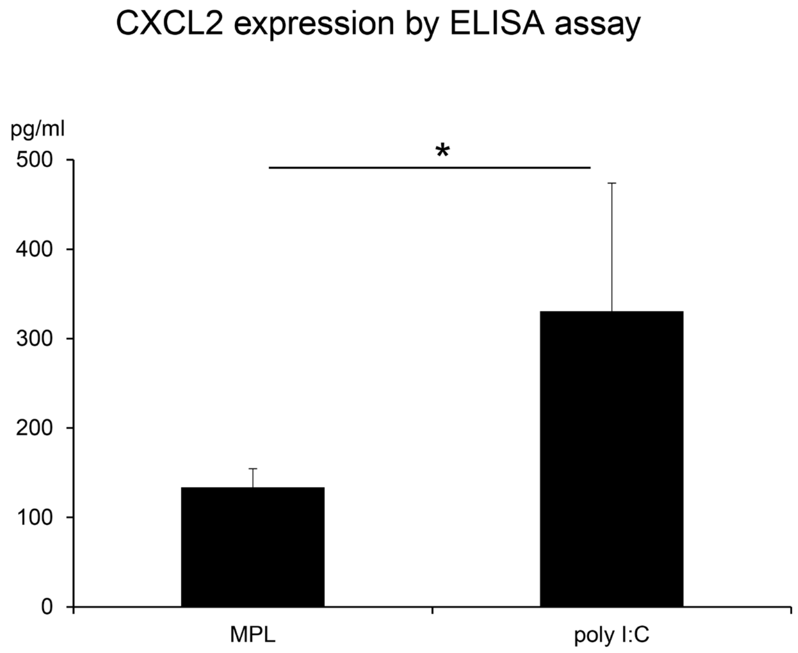

Supplementary Figure S7: CXCL2 expression in tumor tissue after one MPL (10 µg) or three poly I:C (25 µg) treatments was assessed by ELISA, (n=6) *: p<0.05.

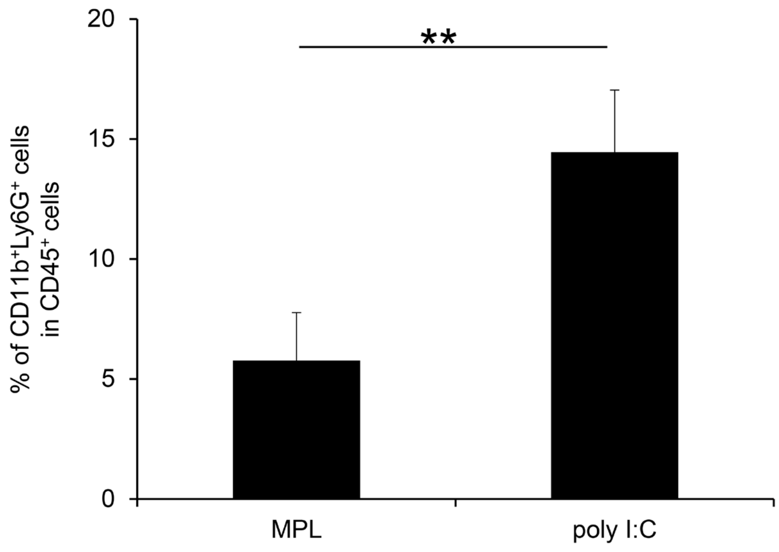

Supplementary Figure S8: FACS analysis of infiltrated neutrophils (CD11b+Ly6G+) in CD45+ cell population in tumors after one MPL (10 µg) or three poly I:C (25 µg) treatments, (n=6). ** Indicates p<0.001.

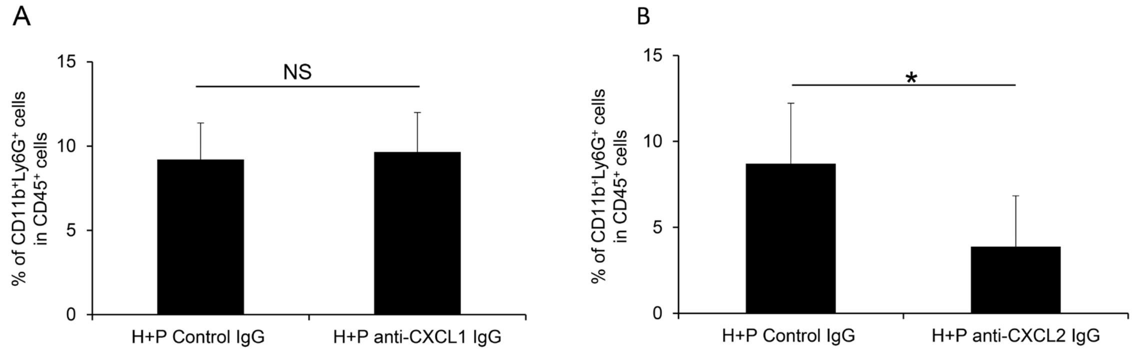

Supplementary Figure S9: Analysis of neutrophil (CD11b+Ly6G+) infiltration after chemokine depletion using A. anti-CXCL1 (5 µg/mouse) or B. anti-CXCL2 (50 µg/mouse) antibodies with HVJ-E+poly I:C (H+P) (25 µg+2500 HAU) treatment in melanoma-bearing mice (n=6) by FACS. As a negative control, an isogenic antibody (Iso) was used. Antibodies were administered intratumorally three times 24 hours before HVJ-E+poly I:C injection and once 24 hours after the treatment (a total of four times). NS: not significant; * Indicates $p<0.05$ (n=7).

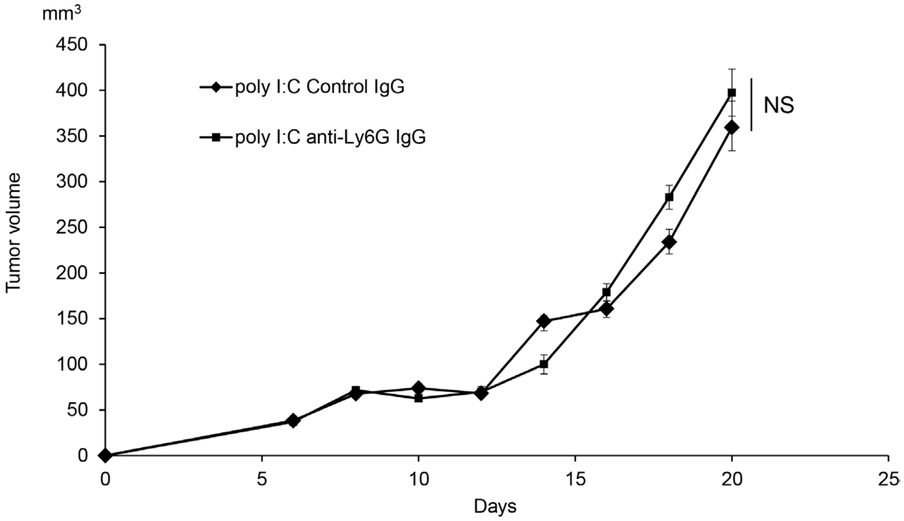

Supplementary Figure S10: Effects of neutrophils on tumor suppression using poly I:C treatment (25 µg) were negligible with and without depletion of neutrophils using the anti-Ly6G antibody (100 µg/mouse) or the isogenic antibody (100 µg/mouse) administered intraperitoneally. NS: not significant.
